# Thermomechanical Properties and Fracture Toughness Improvement of Thermosetting Vinyl Ester Using Liquid Metal and Graphene Nanoplatelets

**DOI:** 10.3390/polym14245397

**Published:** 2022-12-09

**Authors:** Thanh Kim Mai Dang, Mostafa Nikzad, Vi Khanh Truong, Syed Masood, Chung Kim Nguyen, Igor Sbarski

**Affiliations:** 1 School of Engineering, Swinburne University of Technology, P.O. Box 218, Melbourne, VIC 3122, Australia; 2 College of Medicine and Public Health, Flinders University, Adelaide, SA 5042, Australia; 3 School of Engineering, RMIT University, Melbourne, VIC 3001, Australia

**Keywords:** liquid metal, eutectic gallium–indium alloy, graphene nanoplatelets, vinyl ester resin, mechanical properties, fracture toughness

## Abstract

In this study, a eutectic gallium–indium (EGaIn) alloy and graphene nanoplatelets (GnPs) were employed as reinforcements for a comonomer vinyl ester (cVE) resin at different weight fractions up to 2% via a direct polymerization process. First, the effect of EGaIn on the curing kinetics of cVE was evaluated. The thermal and mechanical properties, and the fracture toughness of two types of cVE composites consisting of EGaIn and GnPs were then studied. The results showed that sub-micron sized EGaIn (≤1 wt.%) could promote the curing reaction of cVE without changing the curing mechanism. However, with further increases in EGaIn loading between 1 and 2 wt.%, the curing reaction rate tends to decrease. Both EGaIn and GnPs showed a significant enhancement in strengthening and toughening the cVE matrix with the presence of filler loading up to 1 wt.%. EGaIn was more effective than GnPs in promoting the flexural and impact strength. An increase of up to 50% and 32% were recorded for these mechanical properties, when EGaln was used, as compared to 46%, and 18% for GnPs, respectively. In contrast, the GnPs/cVE composites exhibited a greater improvement in the fracture toughness and fracture energy by up to 50% and 56% in comparison with those of the EGaIn/cVE ones by up to 32% and 39%, respectively. Furthermore, the stiffness of both the EgaIn/cVE and GnPs/cVE composites showed a significant improvement with an increase of up to 1.76 and 1.83 times in the normalized storage modulus, respectively, while the glass transition temperature (T_g_) values remained relatively constant. This work highlights the potential of EGaIn being employed as a filler in creating high-performance thermoset composites, which facilitates its widening applications in many structural and engineering fields, where both higher toughness and stiffness are required.

## 1. Introduction

Vinyl ester resins have been increasingly employed as matrices in composite materials due to their relatively higher modulus, strength, and glass transition temperatures [1,2,3] while maintaining a low molecular weight compared to unsaturated polyester resins. From an industrial perspective, as an intermediate between unsaturated polyesters and epoxies, the cost of these vinyl esters is considerably lower [4], thus making them attractive for large scale applications. Because of their excellent adhesion, ease of processability, rapid setting times, low shrinkage, and chemical resistance [5,6], vinyl esters serve as casting resins, surface coatings [7,8], and high-performance adhesives [9] for a wide range of applications in fields such as the aerospace, automotive, marine, and construction industries. However, the intrinsic brittleness of highly crosslinked thermosets such as vinyl esters limits their use in many structural and engineering fields where toughness and stiffness are important. Local stress concentrations may induce cracks, leading to spontaneous failure because such brittle polymers have poor resistance to crack initiation and propagation [10]. As a result, thermosets with better toughness are always preferred. To improve fracture toughness, additives and fillers such as reactive liquid rubbers [11,12], core shell rubbers [13], block copolymers [14], and stiff inorganic nanoparticles [15,16,17] are frequently used. Despite the success in employing polymeric tougheners, stiff inorganic nanoparticles such as metallic, non-metallic particles, and carbon nanotubes are favoured because they can toughen the matrix without lowering its modulus or glass transition temperature (T_g_) [18,19]. However, a limitation of this approach is that the expected enhancement in the toughness properties of the matrix depends significantly on the uniform dispersion level of these inorganic nanofillers. Moreover, achieving good uniform dispersion at higher nanoparticle volume fractions is challenging due to the poor interaction between the hydrophilic fillers and the hydrophobic matrix [18]. Additionally, particles with high surface energy agglomerate quickly when the particle size decreases [20,21]. Such agglomerates tend to maintain their friable structure in the composite, causing degradation of the mechanical properties of the nanocomposites [22,23]. As a result, despite the fact that ultrasonic dispersion was found to be very effective in preventing the formation of large agglomerates, it was determined that additional chemical and/or mechanical treatment would be required to disperse and deagglomerate nanoparticles in the polymer matrix at higher volume fractions as well as to enhance the composites’ performance [24,25].

In the past decade, substituting rigid fillers with droplets of liquid metals (LMs) to modify polymer matrices has been applied as a promising approach [26,27,28]. LMs with melting points near room temperature such as eutectic gallium–indium (EGaln) alloy (75.5% Ga and 24.5% In) are of particular interest due to their unique features. Apart from having negligible vapour pressure and lower toxicity than mercury [29], a thin oxide shell (1–3 nm) can be easily formed on the surface of a LM droplet in the presence of oxygen [30]. More importantly, this functional native gallium oxide layer allows for the facile formation of stable nanoparticles with high surface areas in aqueous and organic systems via a sonication procedure [31,32,33,34,35]. The physically disrupting concept of passivated metal surfaces is noteworthy since a simple one-step procedure can be applied as an unprecedented opportunity in incorporating LM into polymeric systems. Moreover, low viscosity and high fluidity are the great advantages of LM in injecting it into complex micro-channels. In comparison to rigid inorganic fillers, LM droplets can deform freely with surrounding soft polymers and have an unrestricting effect on elastic compliance and strain limit [36]. Recently, incorporation of micro- and nanoscale EGaIn into elastomers and plastics has been claimed to be a promising approach in producing soft robotics and self-healable electronic devices due to remarkable improvements not only in electrical conductivity and thermal stability, but also in stretchable, flexible, and mechanical properties [37,38]. In this respect, while the use of LMs in improving the performances of composites has been investigated, the thermal and mechanical characteristics of the composites based on LM droplets and thermosets are still lacking in the literature.

Moreover, graphene nanoplatelets (GnPs) have also attracted special attention in reinforcing polymer matrices in recent years due to their outstanding properties and increasingly competitive prices [39,40]. The GnPs powder isolated from pristine graphite is a mixture of a single layer, a few layers, and nanostructured graphite with the thickness varying from 0.34 nm to 100 nm [41]. GnPs are less expensive than carbon nanofibers and nanotubes due to an abundant supply of natural graphite and the ease of manufacture [42]. When GnPs are integrated into polymer matrices, they are also more compatible than other stiff fillers [43]. Moreover, made by the stacking of 2D short graphene platelets, GnPs are unique with a planar structure, high aspect ratio, and large surface area. As a result, GnP-modified polymer composites have shown an effective improvement in a wide range of properties such as mechanical [44,45], gas barrier [46], thermal performance [47], flame retardancy [47,48], and electrical conductivity [49].

This study thus focuses on analysing the effects of different contents of EGaIn on the morphological, thermal, fracture toughness, and mechanical properties of EGaIn-modified comonomer epoxy vinyl ester (cVE) composites (EGaIn/cVE). These thermal and mechanical results were then compared to those of the GnPs-based cVE composites (GnPs/cVE) as a benchmark. The cVE matrix used in this study is an optimum formulation, which has low viscosity, prolonged gel time, and minimal curing time [50]. The cVE was studied based on the incorporation between methyl methacrylate diluent monomer and a commercial vinyl ester polymer. The findings in this work shed light on achieving a novel vinyl ester-based composite with high performance.

## 2. Materials and Sample Preparation

### 2.1. Materials

The polymer used in this study is a commercial vinyl ester resin, Hetron 922^®^ PAW, supplied by Nuplex Composites, a division of Nuplex Industries Pty Ltd., Sydney, Australia. Methyl methacrylate (MMA, 99%) and styrene (ST, 99%) monomers supplied by Chemical Solutions were employed as diluents. Eutectic gallium–indium alloy (EGaIn, 75.5% Ga and 24.5% In, >99%) and methyl ethyl ketone peroxide (MEKP, 99%) were provided by Sigma-Aldrich (Burlington, MA, USA). Graphene nanoplatelets (GnPs) powder with the average surface area of 750 m^2^/g and a diameter of nominally less than 1 µm was provided by XG Sciences (Lansing, MI, USA). Both EGaIn and GnPs were used as reinforcing fillers in this study.

### 2.2. Preparation of EGaIn-Modified Comonomer Vinyl Ester Composites

**Figure 1:**
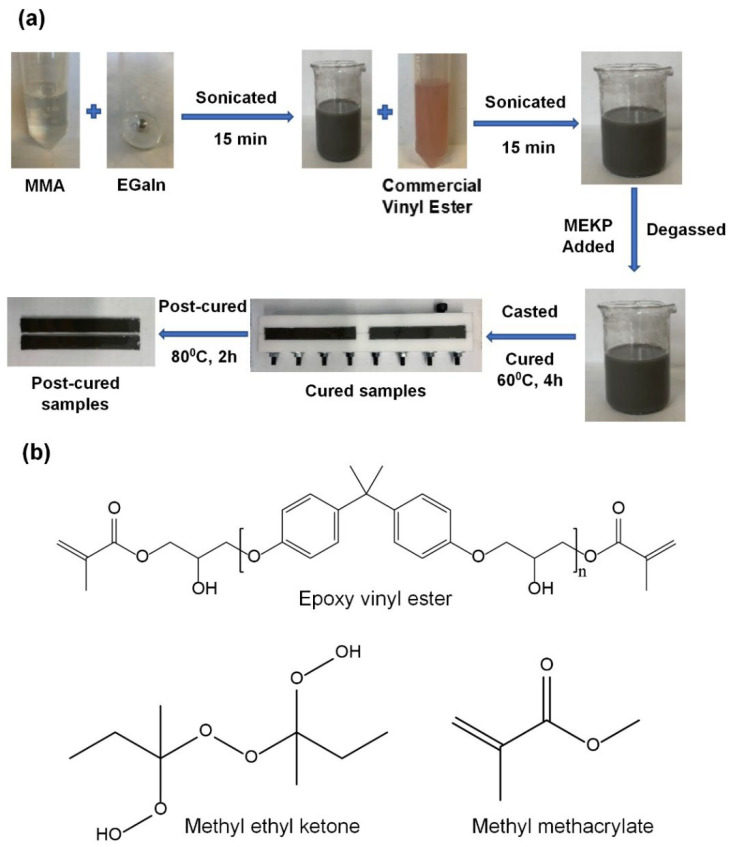
Preparation of EGaIn-modified vinyl ester composites (**a**) and chemical structure of vinyl ester, MEKP, and MMA (**b**).

The processing of the typical test sample preparation of EGaIn-modified comonomer vinyl ester composites (EGaIn/cVE) is outlined in Figure 1a. First, MMA diluent, commercial vinyl ester, and EGaIn at various weight fractions ranging from 0 to 2 wt.% were separately weighed in a fume cupboard. The MMA diluent was then mixed with EGaIn using an ultrasonication technique. A 750 W horn-type ultrasound sonicator (Vibra-Cell, SONICS, Newtown, CT, USA) was employed. Sonication was carried out in an ice bath for 15 min at a frequency of 20% of the maximum power. The commercial vinyl ester was added next. The mixture was further ultrasonicated for 15 min. The total ultrasonication time was 30 min. After mixing with 1.25 wt.% MEKP and degassing under vacuum for 10 min, the composite mixture was poured into the Teflon moulds. They were then cured for 4 h at 60 °C and post-cured at 80 °C for 2 h in a preheated hot air oven to achieve fully cured composite samples. The GnP-filled cVE composite (GnPs/cVE) samples were also prepared in the same procedure as mentioned above. The control samples without EGaIn and GnPs were also prepared. The chemical structures of the vinyl ester, MMA diluent monomer, and MEKP catalyst used in this study are shown in Figure 1b.

## 3. Results and Discussions

### 3.1. Morphological Observation

**Figure 2:**
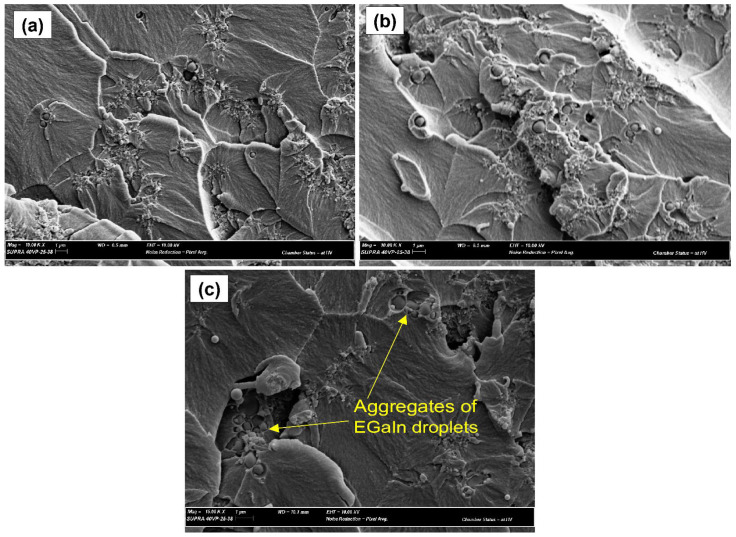
SEM image of the fracture surface of the cVE composites containing 0.5% (**a**), 1%(**b**), and 2% EGaIn (**c**).

**Figure 3:**
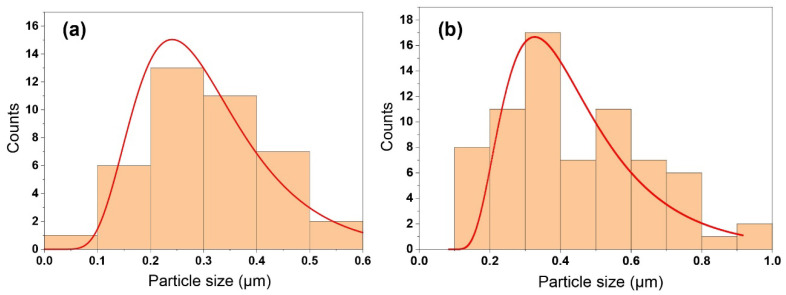
The size distribution of EGaIn droplets within the cVE matrix containing 0.5 wt.% (**a**) and 1 wt.% (**b**).

**Figure 4:**
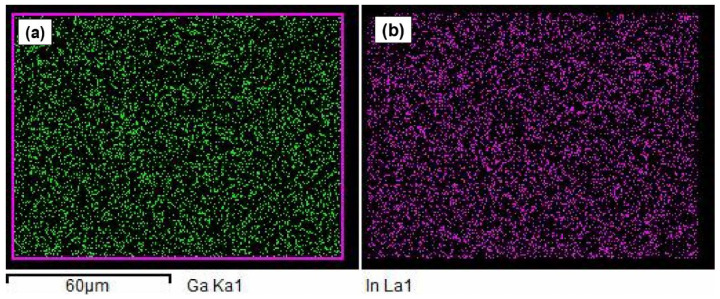
EDX mapping images of the fracture surface of the 1% EGaIn-modified cVE composites. Dispersion of gallium (**a**) and indium (**b**) elements.

The morphology of the reinforced EGaIn filler in the cVE matrix is depicted in Figure 2a–c corresponding to 0.5, 1, and 2.wt.% filler content. It can be seen that EGaIn droplets are in a spherical shape and are discretely dispersed as shown in Figure 2a,b. However, with the incorporation of 2 wt.% filler, some aggregates of EGaIn droplets were observed in Figure 2c. Furthermore, the dispersion and size distribution of EGaIn droplets within the cVE resin were investigated using Image J software based on scanning electron microscopy (SEM) images. It was observed that the size distribution of EGaIn droplets within the cVE matrix of the 0.5 wt.% and 1 wt.% EGaIn-modified cVE composites was in the range of 0.0 to 1.0 µm as shown in Figure 3a,b. The value corresponding to the peak of the log-normal fitting curve was denoted by the mean diameter, which was approximately 0.25 µm for the cVE composites containing 0.5 wt.% EGaIn (Figure 3a) and approximately 0.33 µm for the 1 wt.% EGaIn-modified cVE composites (Figure 3b). The value corresponding to the peak of the log-normal fitting curve was denoted by the mean diameter [51], which is approximately 0.25 µm for the cVE composites containing 0.5 wt.% EGaIn (Figure 3a) and approximately 0.33 µm for the 1 wt.% EGaIn-modified cVE composites (Figure 3b). There is a clear increase in the mean size of 32% when the filler content increased from 0.5% to 1%. The increase in the mean size and the formation of aggregates of EGaIn droplets with the further addition of filler content could be due to an increase in population density of filler for a given resin volume and a constant time of ultrasonication. On the other hand, the energy dispersive X-ray (EDX) mapping images of the fracture surface of the 1% EGaIn/cVE composite (Figure 4) show extensive dispersion of gallium (Figure 4a) and indium (Figure 4b) elements. This confirms that EGaIn droplets were well-distributed throughout the cross section of the composite sample.

### 3.2. Thermal Analysis

**Figure 5:**
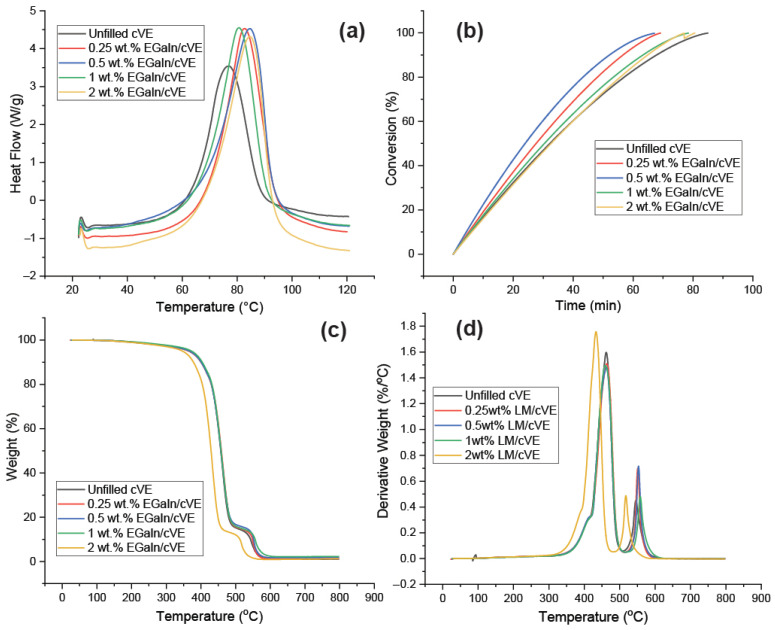
The DSC graphs (**a**), degree of conversion at 60 °C (**b**), graphs of TGA (**d**), and DTG (**c**) at the heating rate of 5 °C/min of the LM filled and unfilled cVE composites.

Differential scanning calorimetry (DSC) and thermogravimetric analyser (TGA) studies were used to investigate the effect of EGaIn on the curing behaviour of cVE networks. Figure 5a depicts modulated DSC heat flow graphs for cVE composites with varying EGaIn concentrations. The curing reaction parameters related to the temperature of onset (T_o_), peak temperature (T_p_), temperature of completion of the exotherm (T_f_), and heat of reaction (ΔH) were noted and are summarised in Table S1 (Supplementary Materials). The initiation of a crosslinking reaction can be seen to be in the range of 65.08–66.94 °C for all of the modified compositions, compared to 65.01 °C for the unfilled cVE system. The same pattern is observed for the completion of exotherm temperature, which is 89.59–92.85 °C for the EGaIn-modified cVE compositions compared to 89.11 °C for the composition without filler loading. It is evident that no significant changes can be observed for the T_o_ and T_f_ values of the filled and unfilled cVE samples. Similar results were also observed for T_p_. In comparison with the unfilled cVE system (76.89 °C), T_p_ values (82.8, 84.9, 80.6, and 84.72 °C corresponding to 0.25, 0.5, 1, and 2 wt.% filler) exhibited an insignificant difference, with the difference being from just 4.8% to 10%. On the other hand, the ΔH values of the EGaIn/cVE are 4.53, 4.52, 4.55, and 4.32 (W/g) corresponding to 0.25, 0.5, 1, and 2 wt.% filler. It can be seen that these values higher by 22% to 27% than those of the composition without filler content (3.54 W/g). The increase in ΔH upon the incorporation of EGaIn suggests a higher cross-linking degree within the cVE matrix, thus implying the effect of EGaIn on the curing reaction. Such behaviour could result from the formation of the ion–dipole interaction between the interface of Ga^3+^ produced from the EGaIn droplets via sonication with the carbonyl group in the cVE polymer chain during the curing process [30]. It should be noted that at higher EGaIn concentrations (2 wt%), the ΔH value shows a slight reduction. This could be explained due to the formation of some aggregates of EGaIn droplets owing to their high concentration, which causes a reduction in activated free radicals, leading to less interaction between EGaIn droplets and cVE matrix. Moreover, the degree of conversion at a certain curing temperature was also employed to determine the curing reaction degree. Figure 5b illustrates the degree of conversion of the filled and unfilled cVE composites at a 60 °C curing temperature. It is apparent that the degree of conversion of the reinforced composites is almost higher than that of the unfilled system except for the degree of conversion of the sample containing 2 wt.% EGaIn, which is nearly the same. It can be seen that this result is consistent with the obtained ΔH result. Figure 5c and d illustrate the thermogravimetric analysis (TGA) and derivative thermogravimetric (DTG) curves of the composites with the content 0.25, 0.5, 1, and 2 wt.% EGaIn. It was observed that the thermograms show the typical two-step degradation of the cVE system as the temperature increases from ambient to 800 °C [52]. As illustrated in Figure 5d, the filled cVE composites containing 0.25, 0.5, and 1 wt.% EGaIn show no significant change in both the decomposition temperature (T_onset_) and T_max_ (the temperatures at maximum weight loss) compared to the unfilled cVE system. This implies that the presence of EGaIn up to 1 wt.% has almost no effect on the thermal stability of the respective EGaIn/cVE composites. However, by adding 2 wt.% EGaIn content, the T_onset_ and T_max_ of this composite decreased and they are both lower than those of the unfilled cVE system. This suggests that further increase in EGaIn loading can promote mass loss during the thermal degradation process.

### 3.3. Thermomechanical Analysis

**Figure 6:**
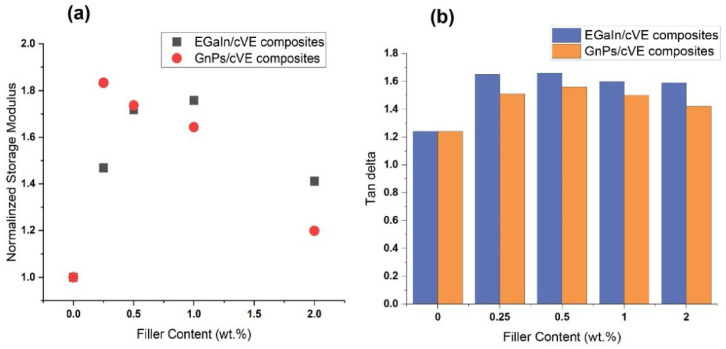
The normalized storage modulus (**a**) and the tan delta (**b**) of the EGaIn/cVE composites and the GnPs/cVE composites with various weight fraction of fillers.

The dynamic properties, including the storage modulus (E′) and tan delta, were determined to investigate the effect of the introduction of fillers on the thermomechanical properties of the cVE matrix. Figure 6 shows the normalized storage modulus and the tan delta of both EGaIn/cVE and GnPs/cVE composites versus the weight fraction of the fillers. The normalized storage modulus was obtained based on the ratio between the storage modulus of the filler-modified cVE composites (E′_EGaIn/cVE_; E′_GnPs/cVE_) and the storage modulus of the cVE matrix (E′_cVE_). As depicted in Figure 6a, both EGaIn/cVE and GnPs/cVE composites exhibit a remarkable enhancement in the normalized storage modulus for all filler contents from 0.25 to 2 wt.%. An increase from 1.4 to 1.76 times in the normalized storage modulus was observed for the EGaIn/cVE composites in comparison with 1.2 to 1.83 times for the GnPs/cVE composites (Table S2, Supplementary Materials). The improvement in the normalized storage modulus of both EGaIn/cVE and GnPs/cVE composites implies that the stiffness of the cVE network would be enhanced as both EGaIn droplets and GnPs were introduced. This phenomenon could be explained by enhancement of the interfacial interactions between the filler interface and the cVE matrix. This effectively restricts the movement of cVE polymer chains with the beginning of glass transition temperature (T_g_) and promotes energy dissipation from the matrix to fillers, resulting in an increase in the elastic modulus [53,54]. However, the improvement of the normalized storage modulus showed a reduction as both EGaIn and GnPs are further added. This behaviour could be attributed to the formation of agglomerations, which limit interfacial contacts between the filler surface and the cVE matrix, resulting in a decrease in the restriction of cVE polymer chain mobility. As a result, the normalized storage modulus enhancement decreased abruptly.

As for tan delta, it can be observed from Figure 6b that both EGaIn and GnPs incorporations into the cVE matrix results in promoting the tan delta of these composites. Moreover, the EGaIn/cVE composites exhibited a greater improvement compared to the GnPs/cVE composites for all filler concentrations from 0.25 to 2 wt.%. The maximum increase in the tan delta of the EGaIn/cVE and GnPs/cVE composites reached approximately 34% and 26%, respectively (Table S2, Supplementary Materials). The improvement in the tan delta suggests that both EGaIn and GnPs incorporation led to an enhanced energy dissipation of their composites. Compared to GnPs nanofiller, the incorporation of EGaIn droplets shows a higher effectiveness in the increase of energy dissipation of the studied composite. Furthermore, there was no significant change in the T_g_ of the EGaIn/cVE composites as EGaIn loading was added. These values varied around 108.7; 109.9; 108.8; and 109.3 °C corresponding to 0.25; 0.5; 1; and 2 wt.% EGaIn loading (Table S2, Supplementary Materials). They are slightly lower from 2 to 3 °C compared to that of the unfilled cVE system (111.8 °C). In comparison to the EGaIn/cVE composites, the T_g_ values of the GnPs/cVE composites shift toward a lower temperature with increasing GnPs content. The obtained T_g_ of the 0.25 wt.% GnPs/cVE composite (111.5 °C) is almost the same as that of the unfilled cVE system (111.8 °C). However, the lower T_g_ values, with them being 110.3; 109.2; and 108.2 °C corresponding to the further addition of EGaIn concentration from 0.25; 0.5; 1; and 2 wt.%, were observed (Table S2, Supplementary Materials). These results are completely consistent with the results obtained from the relationship between the storage modulus of the filler-modified cVE composites (E′_LM/cVE_; E′_GnPs/cVE_) and the storage modulus of the cVE matrix (E′_cVE_). The lower T_g_ phenomenon indicates that, as both EGaIn and GnPs were incorporated, the cross-linked segments of these composites would be more flexible in comparison with that of the cVE matrix.

### 3.4. Mechanical Characteristic

**Figure 7:**
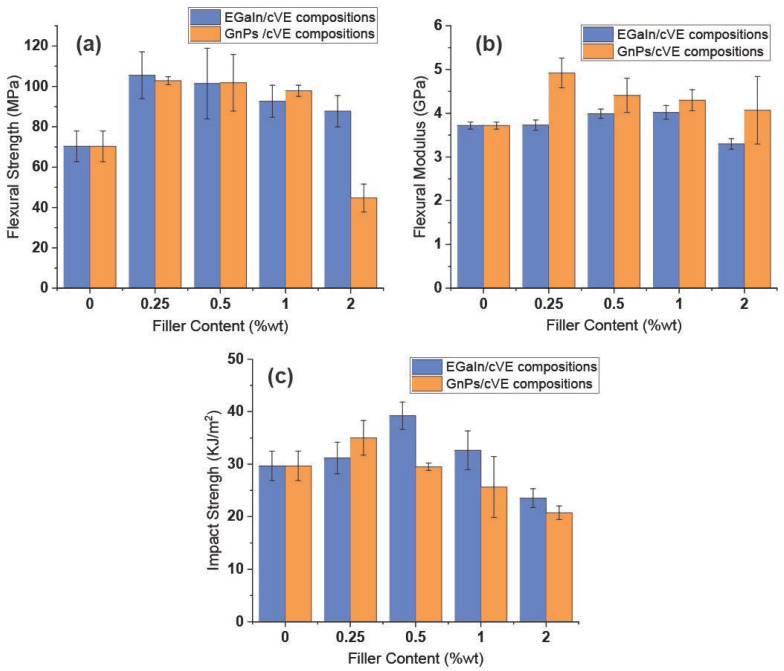
The flexural strength (**a**), flexural modulus (**b**), and impact strength (**c**) of the EGaIn- and GnP-modified cVE composites at different filler contents.

The effects of EGaIn addition on cVE resin flexural characteristics and the impact behaviour were investigated. Figure 7 depicts the mechanical properties of the EGaIn/cVE composites in comparison to those of the GnPs/cVE composites. In Figure 7a, all reinforced composite samples exhibit a remarkable enhancement in flexural strength compared to the case of the unfilled system unless the compositions contain 2 wt.% filler. The flexural strength values of the two types of composites show comparable patterns and remained essentially unaltered at the same filler amount with up to 1 wt.%. The maximum flexural strength of the EGaIn/cVE and GnPs/cVE composites reached 50% and 46% at 0.25 wt.% filler contents, respectively (Table S3, Supplementary Materials). For the EGaIn-modified cVE composites, the flexural strength improvement could be attributed to the strong interfacial interaction between EGaIn and the cVE matrix because of the formation of covalent bonding between the EGaIn surface and cVE polymer chain during the polymerisation process [30,55]. The strong interfacial interaction efficiently promotes the stress transfer from the cVE matrix to the EGaIn fillers, resulting in an increase in the strength performance. Meanwhile for the GnPs/cVE composites, the unique characteristics of GnPs such as a large aspect ratio, high specific surface area, and a planar structure [56], as well as their greater compatibility when incorporated into polymer matrices compared to other rigid nanofillers [57], could be the main factors contributing to the strength improvement of the GnP-modified cVE composites. Because GnPs provide a wider contact area and strong interfacial adhesion with polymer molecular chains, a higher load transfer occurs between the cVE matrix and the GnPs filler. However, as the amount of EGaIn or GnPs filler in the two types of cVE composites grew to 2 wt.%, the flexural strength enhancement benefits rapidly diminished. This behaviour is also observed for the flexural modulus and impact strength of these studied composites, as presented in Figure 7b,c. For the EGaIn-modified cVE composites, this phenomenon could be due to two reasons. The first is the decrease in volume of fine generation EGaIn loading of EGaIn droplets as EGaIn loading increased while the sonication time and input power remained constant. This is because the achieved average size of the dispersed droplets was reported to be proportional to the sonication time and power input and inversely proportional to the processing volume [58]. The second reason is the formation of aggregates of EGaIn droplets for the sample containing 2 wt.% filler loading. The decrease in mean size reduces the overall surface area of EGaIn droplets, but the creation of EGaIn droplet aggregates reduces the interaction between EGaIn droplets and the cVE polymer matrix. Thus, they result in a less efficient stress transfer mechanism, which has a negative impact on the strength properties of EGaIn/cVE composites [56]. Similarly, the decrease in the promotion effects of the flexural and impact strength of the GnP-based cVE composites could be because of the formation of agglomeration when the GnPs content is further increased. This subsequently aggravates in the resins due to weak intermolecular van der Waals forces between low-dimensional carbon nanomaterials [56,59]. These points of agglomeration act as defect sites in the cVE composites, which leads to inhibition of load transfer between the GnPs filler and the cVE matrix, thus resulting in a decrease in the strength performance. Note that the flexural modulus of the composites filled with GnPs always exhibits a larger value than that of the composites filled with EGaIn at the same filler loading levels. The maximum flexural modulus of the composites filled with GnPs is 22% and 32% higher than that of the EGaIn-modified cVE composites and the unfilled one, respectively (Table S3, Supplementary Materials). This indicates that GnPs reinforcement provided greater stiffness enhancement for the cVE matrix compared to EGaIn. Furthermore, the addition of EGaIn improved the impact strength of all EGaIn/cVE composite samples shown in Figure 7c. The same effect is also observed in the impact strength of the 0.25 wt.% GnP-modified cVE sample. These enhancements could be because the propagation of cracks in the cVE resin was limited by the introduction of EGaIn or GnPs fillers. This restriction of crack propagation in both EGaIn- and GnP-modified cVE composites is further discussed by SEM analysis. However, the impact strength of the cVE composites filled with GnPs dropped dramatically with increasing GnPs loading between 0.5 and 2 wt.% (Figure 7c). At 2 wt.% GnPs content, the impact strength value is over 30% lower than that of the unfilled sample (Table S3, Supplementary Materials). This behaviour may be related to the occurrence of agglomeration in the GnPs/cVE composites due to the increase in filler content as discussed above. Note that EGaIn exhibited better reinforcement in enhancing the impact strength of the cVE matrix in comparison with GnPs. The maximum impact strength of the 0.5 wt.% EGaIn/cVE sample exhibited an improvement of 12% and 32% compared to that of the 0.25 wt.% GnPs/cVE sample and the unfilled one, respectively (Table S3, Supplementary Materials).

### 3.5. Fracture Toughness Analysis

**Figure 8:**
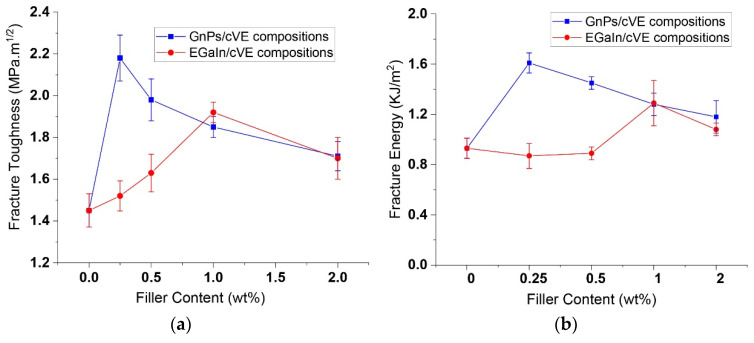
The fracture toughness (**a**) and fracture energy (**b**) of the EGaIn- and GnP-modified cVE composites at different filler contents.

The ability of the cVE resin with and without EGaIn and GnPs fillers in the resistance of the propagation of a pre-existing crack was determined via a Mode I type of fracture failure. The fracture toughness (K_Ic_) and fracture energy (G_Ic_) versus the weight fraction of the fillers are demonstrated in Figure 8. It was observed that the fracture toughness and fracture energy of both EGaIn- and GnP-modified cVE composites were enhanced at all filler loading levels up to 2 wt.%. The GnPs/cVE composites showed a rapid increase in the fracture toughness and fracture energy by up to 50% and 73% at 0.25 wt.% filler content (Table S4, Supplementary Materials), respectively. These enhancements then decreased gradually with further addition GnPs content up to 2 wt.%. The same behaviour was observed for the EGaIn/cVE composites; however, the maximum improvement in the fracture toughness and fracture energy was achieved at 1 wt.% filler content with an increase of over 32% and approximately 39% (Table S4, Supplementary Materials), respectively. Moreover, it can also be seen that EGaIn was less effective than GnPs in promoting the fracture toughness and fracture energy at low filler loadings (under 1 wt.%). The enhancement in the fracture toughness and fracture energy of both EGaIn- and GnP-filled cVE composites may be explained by the fact that the presence of EGaIn or GnPs in the polymer matrix impedes crack propagation, resulting in more efficient energy dissipation and a delay in the onset of failure. The composites therefore sustain a greater amount of applied load. Meanwhile, the reduction in the promotion effects in the fracture toughness and fracture energy of both EGaIn- and GnP-filled cVE composites could be a result of the formation of aggregates of EGaIn droplets and the agglomeration of GnPs within the cVE matrix. Such aggregates and agglomerations act as defect points causing a decrease in the toughening effect owning to the restriction of the interaction between EGaIn and the cVE matrix GnPs or the interfacial adhesion between GnPs and the cVE matrix [56,60].

### 3.6. Fractography Analysis

**Figure 9:**
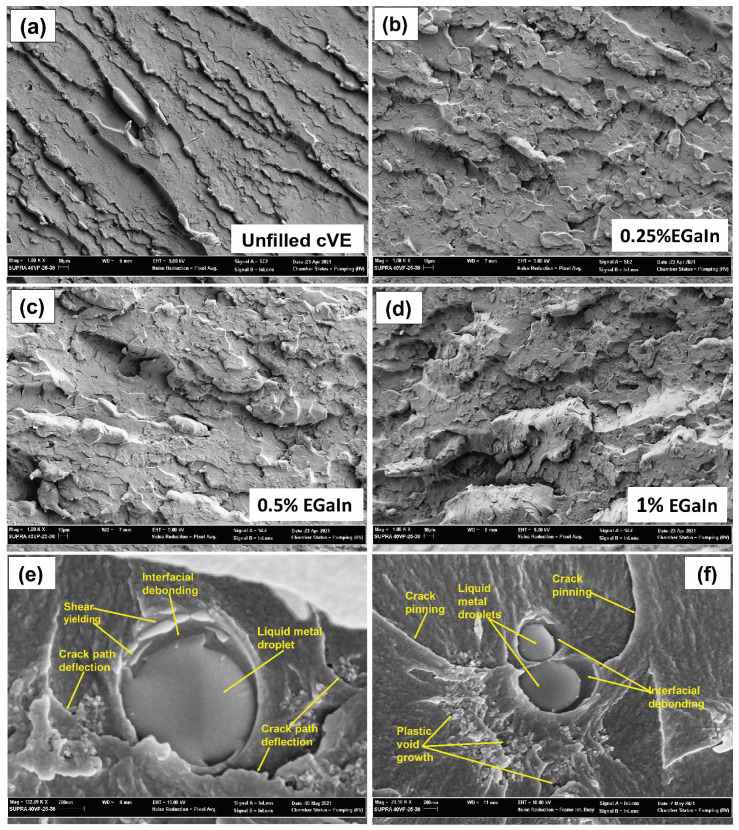
SEM images of the fracture surface of the unfilled cVE matrix (**a**) and the EGaIn/cVE composites, containing 0.25% (**b**), 0.5% (**c**), and 1% (**d**) filler, and the toughness mechanisms in the EGaIn/cVE composites (**e**,**f**).

Figure 9 depicts SEM images of the fracture surface of EGaIn-modified cVE composites. It is evident that the fracture surface of the EGaIn-modified cVE composites is rougher, with many protrusions, and that the area of slow crack propagation is larger than that of the unfilled resin composites (Figure 9a–d). This implies that the presence of EGaIn in the cVE matrix can transform the cracking process from a brittle cracking to a discontinuous cracking process, which is clear evidence of the toughness enhancement of the EGaIn-modified cVE composites. Moreover, the interfacial debonding between EGaIn and the cVE matrix also occurred in the EGaIn/cVE composites as shown in Figure 9e,f This phenomenon is recognized as an efficient intrinsic toughening mechanism for composite materials since it acts as a trigger for additional energy-absorbing mechanisms, such as plastic yielding (Figure 9e) and the formation of plastic voids (Figure 9f), due to the stress concentrations caused by the reinforced filler’s high stiffness [61,62]. Furthermore, the behaviour of the changing direction of the cracks with some irregular crack edges around the EGaIn droplet can be seen in Figure 9e. This is because the moving cracks’ front is forced out of the initial propagation plane when they meet an impenetrable obstacle, which is a feature of the crack-path deflection mechanism [63]. Moreover, the propagating crack front could also be pinned and forced to bow out when facing an array of EGaIn droplets. The interparticle crack length increases until the crack front breaks away at a critical state, forming local semicircular cracks as shown in Figure 9f. This behaviour is known as the crack pinning mechanism [64]. These events result in absorption of more energy to propagate the cracks due to the creation of additional crack surfaces. This confirmed the enhancement in the fracture energy and fracture toughness of the EGaIn/cVE composites. In addition, the increase in the filler reinforcement is accompanied by the increase in the density of microcracks and micro-voids within the process zone when the spacing between particles is sufficiently large and the fillers are evenly dispersed [65,66]. This may contribute to a quick increase in the fracture energy and fracture toughness values of the EGaIn/cVE composites up to a content of 1 wt.% as seen in Figure 9. However, the formation of some aggregates of EGaIn droplets with the further addition of EGaIn content to 2 wt.%, as shown earlier in Figure 2c, could be evidence of the reduction in the toughening effects of the EGaIn/cVE composites as mentioned above.

**Figure 10:**
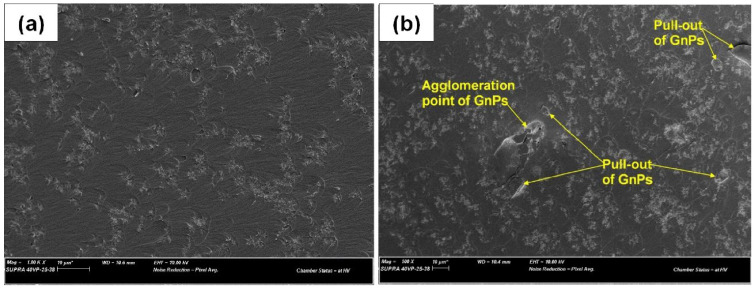
SEM micrograph of the fracture surface of the 0.25% GnPs/cVE composite (**a**) and 0.5% GnPs/cVE composite (**b**).

The SEM images of the fracture surface of the GnP-modified cVE composites are shown in Figure 10. In comparison with the composites containing 0.25 wt.% EGaIn (Figure 10a), the 0.25% GnPs/cVE composite exhibits a smoother fracture surface as well as GnPs particles interacting with the cracks in the form of small aggregates (Figure 10a). This could be because of the difference in size and geometrical features between GnPs and EGaIn. At the same volume fraction, GnPs (nano scale) provide a higher interfacial surface area with the cVE matrix than that provided by EGaIn (sub-micron scale). This results in a substantially lower interfacial stress concentration in the composites reinforced by GnPs due to a reduction in interparticle spacing, leading to further mitigation of the crack initiation [65]. In term of this, it was reported that the crack pinning mechanisms on the fracture surfaces of the composites reinforced by nanofillers were much less prominent than those modified by micro-fillers with well dispersed fillers [66,67]. Moreover, the pull-out of GnPs platelets from the cVE polymer matrix is also observed in Figure 10b. The pull-out event can slow crack propagation and induces energy dissipation when the crack opens due to the friction between GnPs and the cVE matrix, resulting in a limitation of crack expansion and enhancement of the fracture toughness. However, a small amount of agglomeration is also observed in the 0.5% GnPs/cVE composite (Figure 10b). Such agglomerations act as defect points, which are easily broken or misshapen because the interfacial adhesion between GnPs and the cVE matrix is obviously limited. This observed result confirms a reduction in both the strengthening and toughening effect of the GnPs/cVE composites as mentioned above. It should be also noted that in the cases of filler concentrations under 1 wt.%, both the fracture toughness and fracture energy of the EGaIn/cVE samples are lower than those of the GnPs/cVE samples at the same filler loading levels, as was shown earlier in Figure 8. The difference in these enhancement effects could be attributed to the size and geometrical feature differences between GnPs and EGaIn. As discussed in this session, 2D nanoplates (GnPs) provide a larger interface area and lower interparticle spacing than the 1D EGaIn droplets, which yield greater energy dissipation, resulting in higher toughening enhancement for the cVE composites reinforced by GnPs. In addition, the difference in interfacial interactions of micro- and nanofillers enveloped by the cVE matrix could be another reason affecting the distinct enhancements of the thermomechanical properties and fracture toughness of the EGaIn- or GnP-modified cVE composites. With a higher surface area and a higher number density compared to EGaIn (sub-micro-filler) at an equal volume fraction in a well dispersed state, GnPs (nanofiller platelets) possess larger numbers of overall interfacial interaction regions. This behavior results in greater transportation of load at the interfacial interaction zone due to a higher area of stress transferring regions [56]. Thus, the improvements in the thermomechanical properties and fracture toughness of the GnPs/cVE composites was more effectively promoted.

## 4. Conclusions

A unique thermoset composite was created by incorporating EGaIn into a comonomer epoxy vinyl ester resin through direct polymerization. The effects of EGaIn on the curing kinetics and thermal characteristics of the cVE matrix were examined in this study. In addition, the fracture toughness and the dynamic and static mechanical properties of two types of EGaIn- and GnP-modified cVE composites were examined. The obtained results indicate that the curing reaction of the cVE resin could be promoted without changing the curing mechanism. However, the curing reaction rate tends to decrease and the mass loss during the thermal degradation process could be promoted with further increases in EGaIn loading to 2 wt.%. The flexural and impact strength of the EGaIn/cVE and GnPs/cVE composites were improved significantly compared to that of the unfilled cVE system. For the EGaIn/cVE composites, the improvement in the flexural and impact strength reached 50% with 0.25 wt.% EGaIn loading and 32% with 0.5 wt.% loading, respectively. Compared to the EGaIn/cVE composites, the GnPs/cVE composites showed a relative similarity in the improvement of flexural strength (46% with 0.25 wt.% filler content), but a lower effectiveness in the enhancement of impact strength (18%). In contrast, the GnPs/cVE composites exhibited a larger improvement in the fracture toughness and fracture energy by up to 50% and 56% in comparison with those of the EGaIn/cVE ones by up to 32% and 39%, respectively. Additionally, an increase up to 1.76 and 1.83 times in the normalized storage modulus as well as 34% and 26% in the tan delta were recorded for both the EGaIn/cVE and GnPs/cVE composites, respectively, whereas the glass transition temperature (T_g_) values remained relatively constant. As a result, incorporating EGaIn into cVE resin provided a noticeable improvement in the mechanical properties as well as in the enhanced toughness and stiffness of the cVE matrix without degrading its thermal properties. This simple and effective approach thus expands the applications of thermoset composites in many structural and engineering fields where both toughness and stiffness are critical. Our team’s investigations in this research field are continuing and further outcomes will be reported in future publications.

## Data Availability

Data available within the article and its Supplementary Materials or from the authors upon reasonable request.

## Supplementary Materials

The following supporting information can be downloaded at: https://www.mdpi.com/article/10.3390/polym14245397/s1, Table S1: Results of DSC scans of the EGaIn/cVE composites at different filler contents; Table S2: Results of storage modulus (E’), normalized storage modulus, Tan delta and glass transition temperature (Tg) of both EGaIn/cVE and GnPs/cVE composites at different filler contents; Table S3: The flexural strength (σf), flexural modulus (Ef) and impact strength (αC) of the fillers modified and unmodified cVE composites; Table S4. The fracture toughness (KIc) and fracture energy (GIc) of the fillers modified and unmodified cVE composites. References [68,69,70,71] are cited in the supplementary materials.
