# Supplementary material for: Thermomechanical Properties and Fracture Toughness Improvement of Thermosetting Vinyl Ester Using Liquid Metal and Graphene Nanoplatelets
Source: Polymers (Basel). 2022 Dec 9;14(24):5397. doi: 10.3390/polym14245397 (PMC9783833; doi:10.3390/polym14245397)
Supplement: Supplementary file 1 [file polymers-14-05397-s001.zip › polymers-2011686-supplementary.pdf]

polymers MDPI

Supplementary Materials

Thermomechanical Properties and Fracture Toughness Improvement of Thermosetting Vinyl Ester Using Liquid Metal and Graphene Nanoplatelets

Thanh Kim Mai Dang[a], Mostafa Nikzad[a*], Vi Khanh Truong[b], Syed Masood[a], Chung Kim Nguyen[c] and Igor Sbarski[a]

[a]School of Engineering, Swinburne University of Technology, PO Box 218, Melbourne, VIC, 3122, Australia
[b]College of Medicine and Public Health, Flinders University, GPO Box 2100, Adelaide, 5001, Australia
[c]School of Engineering, RMIT University, Melbourne, VIC, 3001, Australia

Methods and Tables of data

1. Thermal Property Measurements

The curing behaviour and the thermal stability of the eutectic gallium-indium (EGaIn) reinforced and unreinforced cVE composites were measured using differential scanning calorimetry (DSC) model 2920 TA instruments and a thermogravimetric analyser (TGA). For DSC experiments, a sample size of 5 to 10mg was ramped at 5°C/min from room temperature to 120°C in a nitrogen atmosphere, while for TGA measurements, a sample size of 10 to 15mg was heated from room temperature to 800°C at a heating rate of 10°C/min in a nitrogen atmosphere. The dynamic mechanical properties of the fillers modified and unmodified cVE composites were carried out using model Q800 TA instruments as per ASTM D7028-7 [1]. The uniform rectangular specimens of 45 mm × 12 mm × 3 mm were prepared. These samples were tested on three-point bending mode at frequency 1Hz with a deflection of 30 μm with a temperature ramp from 25°C to 150°C at a heating rate of 3°C/min.

2. Fracture Toughness

A single-edge notched three-point flexural test (SENB) was employed to determine the fracture toughness according to the ASTM D5045-99 standard [2]. Rectangular specimens of 26.4mm x 6mm x 3mm with a 3mm long notch were prepared for the measurement. The specimens were fixed to the testing machine Z010/TN2S (Zwick, Germany), and a load of 10 mm/min was applied. The critical stress intensity factor (K_{Ic}) was adopted to inquire into the fracture toughness. The value of K_{Ic} in is determined based on the ASTM D5045-99 standard as follows:

$$K_{Ic} = \left(\frac{P}{Bw^{\frac{1}{2}}}\right) f(x) \tag{S1}$$

where K_{Ic} is the critical stress intensity factor in MPa.m$^{1/2}$, P is the loading weight in Newtons, B is the thickness of the specimen in mm, w is the width of the specimen in mm, a is the crack length in mm, x is the ratio of the crack length to the depth of specimen, a/w, and f(x) is the calibration factor, which is represented as follows:

$$f(x) = x^{1/2}\frac{[1.99 - x(1-x)(2.15 - 3.93x + 2.7x^2)]}{(1-2x)(1-x)^{3/2}} \tag{S2}$$

3. Impact Strength

The Izod impact test was used to determine the impact strength according to the ASTM D25 [3]. This test was run on the unnotched specimens with dimensions of 72mm x 12.7mm x 3.2mm. The impact strength was calculated using equation (S3).

$$\alpha_C = \frac{E_C}{b.d} \quad \text{(S3)}$$

where α_c is the Charpy impact strength in kJ/m^2, E_C is impact resistance in J/m, b is the measured specimen width in mm and d is the measured specimen thickness in mm. The flexural strength of hardened, filed, and unfilled cVE composite specimens with dimensions of 60mm × 12mm × 3mm was measured on a testing machine Z010/TN2S (Zwick, Germany).

4. Mechanical Properties

The flexural strength of hardened, filed, and unfilled cVE composite specimens with dimensions of 60mm × 12mm × 3mm was measured on a testing machine Z010/TN2S (Zwick, Germany). Three-point bending test with a crosshead speed of 2.5 mm/min and a span length of 48mm was applied according to ASTM D7264 [4]. The flexural strength was calculated using equation (S4) as follows:

$$\sigma_f = \frac{3P_fL}{2bd^2} \quad \text{(S4)}$$

where σ_f is the flexural strength in MPa), P_f is the maximum load at failure in Newtons, L is the support span in mm, b is the measured specimen width in mm and d is the measured specimen thickness in mm.

5. Scanning Electron Microscopy (SEM) Analysis

The scanning electron microscopy (SEM), Suppra 40VP (ZEIZZ) were used to analyse toughness mechanisms of filled and unfilled composite samples. The distribution of LM within the polymer matrix also was determined by the same instrument in SEM-EDX mode. Electron beam operated at 20kV was used in the Energy Dispersive X-Ray spectroscopy, which has been calibrated through standard SiO_2 sample at 50,000 cps. All samples were coated with a thin layer of gold by high-vacuum evaporation before observing the fracture cross sections to avoid charging under the electron beam and viewed perpendicularly.

Table S1. Results of DSC scans of the EGaIn/cVE composites at different filler contents.

Filler Content (wt.%)	T_0(°C)	T_p(°C)	T_f(°C)	$\Delta H \left(\frac{W}{g}\right)$
0	65.01	76.89	89.11	3.54
0.25	65.08	82.77	89.59	4.53
0.5	66.30	84.90	88.99	4.52
1	65.60	80.64	91.39	4.55
2	66.94	84.72	92.85	4.32

Table S2. Results of storage modulus (E′), normalized storage modulus, Tan delta and glass transition temperature (T_g) of both EGaIn/cVE and GnPs/cVE composites at different filler contents.

Filler Content (wt.%)	EGaIn Compositions				GnPs Compositions			
	E′(GPa)	Normalized Storage Modulus	Tan delta	$T_g(°C)$	E′(GPa)	Normalized Storage Modulus	Tan delta	$T_g(°C)$
0	9.73	1	1.24	111.8	9.73	1	1.24	111.8
0.25	14.29	1.47	1.65	108.7	17.84	1.83	1.50	111.5
0.5	16.72	1.72	1.66	109.9	16.9	1.74	1.56	110.3
1	17.11	1.76	1.60	108.8	15.99	1.64	1.50	109.2
2	13.73	1.41	1.59	109.3	11.66	1.20	1.42	108.2

Table S3. The flexural strength (σ_f), flexural modulus (E_f) and impact strength (α_C) of the fillers modified and unmodified cVE composites.

Filler Content (wt.%)	EGaIn Compositions			GnPs Compositions		
	$\sigma_f(MPa)$	E_f(GPa)	$\alpha_C(KJ/m^2)$	$\sigma_f(MPa)$	E_f(GPa)	$\alpha_C(KJ/m^2)$
0	70.3±7.6	3.72±0.08	29.67±2.8	70.3±7.6	3.72±0.08	29.67±2.8
0.25	105.5±11.6	3.73±0.12	31.16±3.0	102.8±2.0	4.92±0.44	35.0±3.3
0.5	101.4±17.5	3.99±0.11	39.22±2.6	101.8±1.4	4.41±0.39	29.5±0.7
1	92.6±7.9	4.02 ± 0.16	32.62±3.7	97.8±2.8	4.30±0.24	25.6±5.8
2	87.7±7.8	3.3±0.12	23.51±1.8	44.7±6.9	4.07±0.77	20.7±1.3

Table S4. The fracture toughness (K_{Ic}) and fracture energy (G_{Ic}) of the fillers modified and unmodified cVE composites.

Filler Content (wt.%)	EGaIn Compositions		GnPs Compositions	
	$K_{IC}(MPa.m^{1/2})$	$G_{IC}(KJ/m^2)$	$K_{IC}(MPa.m^{1/2})$	$G_{IC}(KJ/m^2)$
0	1.45 ± 0.08	0.93 ± 0.08	1.45 ± 0.08	0.93 ± 0.08
0.25	1.52 ± 0.07	0.87 ± 0.1	2.18 ± 0.11	1.61 ± 0.08
0.5	1.63 ± 0.09	0.89 ± 0.05	1.98 ± 0.1	1.45 ± 0.05
1	1.92 ± 0.05	1.29 ± 0.18	1.85 ± 0.05	1.28 ± 0.09
2	1.7 ± 0.10	1.08 ± 0.05	1.71 ± 0.07	1.18 ± 0.13

References

1. D-07, A., *Standard test method for glass transition temperature (DMA Tg) of polymer matrix composites by dynamic mechanical analysis (DMA)*. 2015, ASTM International West Conshohocken, PA.
2. ASTM, I., *Standard test methods for plane-strain fracture toughness and strain energy release rate of plastic materials.* ASTM D5045-99, 2007.
3. International, A., *Standard test methods for determining the izod pendulum impact resistance of plastics*. 2010: ASTM international.
4. Testing, A.S.f. and Materials. *ASTM D7264: Standard Test Method for Flexural Properties of Polymer Matrix Composite Materials*. 2015. American Society for Testing and Materials.
